# Targeting dopamine receptor D3 signalling in inflammation

**DOI:** 10.18632/oncotarget.14601

**Published:** 2017-01-12

**Authors:** Rodrigo Pacheco

**Affiliations:** Departamento de Ciencias Biológicas, Facultad de Ciencias Biológicas, Universidad Andres Bello, Santiago, Chile; Laboratorio de Neuroinmunología, Fundación Ciencia & Vida, Ñuñoa, Santiago, Chile

**Keywords:** T-cell mediated immunity, dopaminergic system, dopamine receptors, neuroinflammation, inflammatory disorders, Neuroscience

During last 15 years dopamine has emerged as a major regulator of inflammation. All five dopamine receptors (DRs, DRD1-DRD5) have been found to be expressed in immune cells where they exert a complex regulation of immunity [1]. Of note, DRs have been found not only in cells of the adaptive immune system, but also in cells belonging to the innate immunity, even including glial cells. The outcome of the dopamine effect in the immune response depends in many factors, including differential expression of DRs in the immune cells present in the inflamed tissue, the local levels of dopamine and the signalling coupled to and the affinity of the different DRs involved. An increasing number of studies analysing human cells *in vitro* or using *in vivo* approaches in animal models have been unravelling and deciphering the complexity of dopaminergic regulation of immunity. Integrating the knowledge acquired by these studies, the evidence have indicated that stimulation of low-affinity DRs, for instance DRD1 and DRD2, are coupled to anti-inflammatory mechanisms, thereby dampening inflammation [2, 3]. Conversely, signalling triggered by high-affinity DRs, including DRD3 and DRD5, have been found consistently to promote inflammation [4, 5].

It is noteworthy that tissues containing high-levels of dopamine in steady-state, such as the nigrostriatal pathway or the gut mucosa, undergo a strong decrease of dopamine levels during inflammation [1]. This fact involves a switch in the stimulation of DRs: low-affinity DRs, which display anti-inflammatory properties and are stimulated by high dopamine levels under homeostasis, are not longer stimulated during inflammatory processes. Otherwise, signalling coupled to high-affinity DRs, which seems to be dampened by low-affinity DRs stimulation under homeostasis, becomes dominant when dopamine levels are reduced during inflammation. This idea highlight the relevance of high-affinity DRs favouring the development and progression of inflammatory disorders and makes these receptors key therapeutic targets.

**Figure 1:**
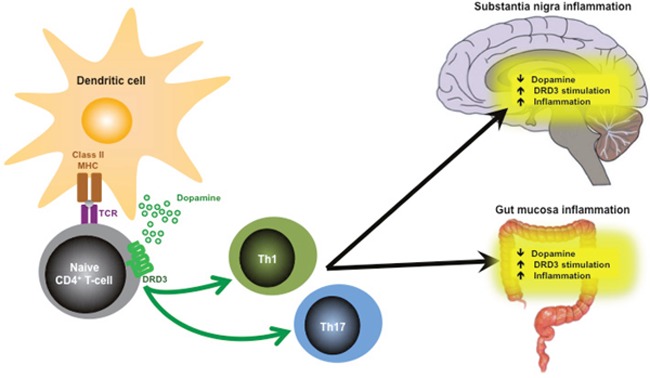
Role of DRD3-signaling in CD4^+^ T-cells favouring Th1 and Th17 mediated responses in disorders involving reduction of dopamine levels in the target tissue, such as Parkinson’s disease and inflammatory bowel diseases.

Accordingly, DRD3, which display the highest affinity by dopamine, has been strongly involved in favouring inflammation in several experimental systems. In this regard, genetic and pharmacological evidence has indicated that DRD3-signalling constitutes a potent regulator of CD4^+^ T-cell-mediated responses, including those implicated in Parkinson’s disease [6] and inflammatory colitis [4], two pathologies that involve a reduction of dopamine levels in the inflamed tissue (Figure 1). Mechanistic analyses have revealed that DRD3-signalling in CD4^+^ T-cells induces suppressor of cytokine signalling 5 in these cells, thus attenuating T-helper 2 (Th2) differentiation and promoting Th1 responses. Moreover, evidence has also indicated that DRD3-signalling favours Th17-immunity under chronic inflammatory conditions [4]. According to the pivotal role of Th1 and Th17 inflammatory responses in the development of Parkinson’s disease, we have recently demonstrated the therapeutic potential of DRD3- antagonism in two different animal models, including 6-hydroxydopamine-induced and 1-methyl-4-phenyl- 1,2,3,6-tetrahydropyridine-induced Parkinson’s disease [7]. In those studies, DRD3-antagonism not only reduced the neurodegenerative and neuroinflammatory process, but also attenuated significantly the motor impairment associated to the loss of dopaminergic neurons.

Importantly, the therapeutic effect observed in Parkinson’s disease models in our studies [7] was exerted by a highly-selective DRD3 antagonist, PG01037, displaying the Ki values 0.70, 93.3 and 375 nM for DRD3, DRD2 and DRD4 respectively. In apparent controversy with our recent findings, pramipexole, a drug described as a DRD3-agonist, has been used for the symptomatic treatment of Parkinson’s disease. However, the range of DRD3-selectivity for this drug is very limited, displaying Ki values of 0.5, 3.3, 3.9 and 3.9 nM for DRD3, DRD2S, DRD2L and DRD4 respectively. Thereby, it is likely that at therapeutic concentrations pramipexole stimulates the dominant effects of DRD2-signalling, abolishing DRD3- mediated effects.

Interestingly, the therapeutic effect of DRD3- antagonism seems to be beyond of targeting DRD3 confined to CD4^+^ T-cells. Our analyses performed in glial cells have suggested that DRD3-deficiency gives an anti-inflammatory behaviour to astrocytes, which results in attenuated microglial activation in a model of Parkinson’s disease [7]. Furthermore, recently we found that DRD3-deficiency or DRD3-antagonism in dendritic cells results in an exacerbated stimulation of CD8^+^ T-cell-mediated immunity, strengthening the immune response against tumours [8]. Mechanistic analyses indicated that the inhibition of DRD3-signalling in dendritic cells promotes an increase in antigen cross-presentation in class I MHC molecules to CD8^+^ T-cells, thereby potentiating the development of cytotoxic T-lymphocytes. Thus, emerging evidence indicates DRD3 as a key therapeutic target with a dual potential: whereas DRD3-inhibtion attenuates inflammation in pathologies associated to reduction of dopamine levels and CD4^+^ T-cell-mediated responses (Figure 1), blocking DRD3-signalling in dendritic cells may improve the outcome of disorders that involve insufficient cytotoxic T-lymphocyte-responses, such as cancer.
